# Continuous and noninvasive respiratory effort monitoring: a narrative review of emerging techniques

**DOI:** 10.1186/s40560-026-00852-z

**Published:** 2026-01-21

**Authors:** J. Graßhoff, R. S. P. Warnaar, D. W. Donker, P. Rostalski, E. Oppersma

**Affiliations:** 1 Fraunhofer Research Institution for Individualized Medical Technology and Engineering IMTE, Mönkhofer Weg 239a, 23562 Lübeck, Germany; 2 Cardiovascular and Respiratory Physiology, Technical Medical Centre, University of Twente, P.O. Box 217, 7500 AE Enschede, The Netherlands; 3 Intensive Care Centre, University Medical Centre Utrecht, Postbus 85500, 3508 GA Utrecht, The Netherlands; 4 Institute for Electrical Engineering in Medicine, University of Lübeck, Moislinger Allee 53-55, Building 19, 23558 Lübeck, Germany

**Keywords:** Diaphragm-protective ventilation, Work of breathing, Airway occlusion maneuvers, Surface electromyography, Model-based estimation, Ultrasound

## Abstract

Ventilator-induced lung injury and diaphragm dysfunction are well-recognized complications of mechanical ventilation and commonly attributed to inadequate ventilator settings. Excessive or insufficient assistance and the patient’s own respiratory effort are increasingly acknowledged as important factors in the pathogenesis of these injuries. Therefore, monitoring respiratory effort at the bedside is a highly relevant strategy to identify and prevent potentially injurious breathing patterns. Esophageal manometry remains the reference standard for assessing respiratory effort, but its technical complexity limits routine clinical use. Placement and calibration of the esophageal balloon are time-consuming and require specific expertise. Moreover, the invasive nature of the procedure precludes visual confirmation and leads to uncertainty about correct positioning, reducing confidence in the validity of measurements. Innovative noninvasive and continuous monitoring techniques are emerging as more accessible and scalable alternatives, enabling assessment of respiratory effort without impacting so much on clinical workflow. This narrative review provides an in-depth overview of three noninvasive techniques that are reshaping continuous respiratory effort monitoring: (1) Surface electromyography (sEMG) now enables continuous monitoring of respiratory muscle activity and derivation of continuous effort estimation using electrodes placed on the torso of the patient. (2) Computational modeling offers dynamic, patient-specific effort estimation from ventilator waveforms. (3) Assessment of diaphragm thickening fraction, derived from high-resolution ultrasound, provides a straightforward surrogate for effort, driven by widely available acquisition devices. Together, these innovations promise to make respiratory muscle monitoring less labor-intensive and more clinically sustainable—paving the way for broader implementation of diaphragm-protective ventilation strategies in critical care.

## Introduction

Mechanical ventilation (MV) provides lifesaving support for patients with acute and chronic respiratory failure. However, MV contributes to ventilator-induced lung injury (VILI) and diaphragm dysfunction (VIDD), especially in the presence of excessive or insufficient respiratory effort. During assisted modes of ventilation, maintaining spontaneous breathing can help preserve diaphragm function and improve patient–ventilator interaction. Yet, recent studies showed that both insufficient and excessive breathing effort are associated with diaphragm dysfunction [1–3]. To minimize harm to both lungs and diaphragm, spontaneous effort during MV should be carefully regulated to remain within a safe and physiologically appropriate range, while asynchronous breaths should be avoided [4].

To assess respiratory effort in daily clinical practice, occlusion methods have been validated as simple, noninvasive bedside techniques [5, 6]. While these techniques appear relatively easy to perform and are commonly used in ventilated patients, their interpretation poses significant challenges. A key limitation of occlusion techniques is that they provide only intermittent measurements of respiratory effort, rather than continuous monitoring. As a result, temporal trends and significant fluctuations in respiratory effort over time may be overlooked. Patient–ventilator asynchrony, which is characterized by a temporal mismatch between the neural inspiratory time of the patient and support from the ventilator, may not be identified [6, 7].

Continuous monitoring of respiratory effort can currently only be realized at the bedside using esophageal pressure (*P*_es_) measurement, which is still considered the reference standard. The total pressure generated by the respiratory muscles (*P*_mus_) can be derived from *P*_es_ by correcting for chest wall elastance [8]. The value of *P*_mus_ reflects the combined effort of the diaphragm and accessory inspiratory muscles, offering a comprehensive and continuous assessment of total respiratory effort. Despite its physiological accuracy, *P*_es_ monitoring faces several practical limitations that prevent its widespread clinical use. The procedure is invasive and requires meticulous catheter placement, optimal balloon inflation, and calibration maneuvers, all of which require very specific expertise. Notably, the interpretation of *P*_es_ waveforms can be cumbersome due to potential artifacts such as cardiac oscillations or esophageal spasms, and the absence of direct visual feedback on the catheter’s precise position further complicates analyses. Consequently, *P*_es_ monitoring remains largely confined to research settings and specialized centers, and a more widespread adoption in routine clinical practice remains limited.

An alternative to assessing the mechanical output of respiratory muscles [9] is a measurement of the electrical field generated by muscles during contraction, the so-called electromyogram (EMG). The current standard technique for measuring the electrical activity of the diaphragm is based on similar nasogastric catheters as used for *P*_es_ monitoring, but featuring electrodes positioned at the level of the diaphragm. It shares potential disadvantages with *P*_es_ monitoring as it also requires a correct placement of a nasogastric catheter.

Given the limitations of bedside maneuvers that can only very intermittently be deployed and the complex and invasive reference methods for continuous assessment, there is a clear need to advance noninvasive approaches that enable real-time monitoring of respiratory effort.

**Fig. 1:**
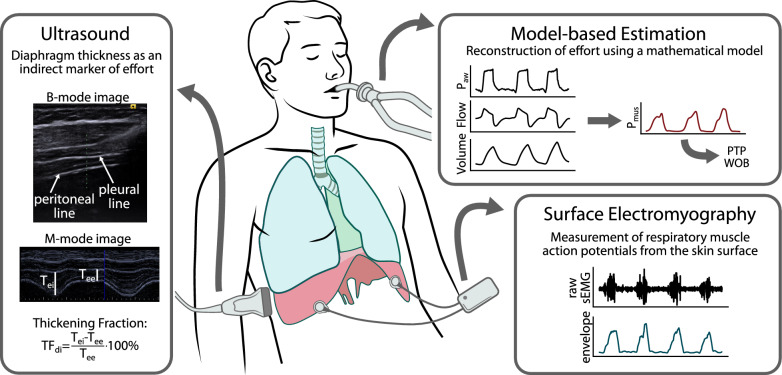
Overview of potential methods for noninvasive and continuous monitoring of respiratory muscle effort. Surface electromyography (sEMG) captures the electrical activity of respiratory muscles using electrodes placed on the skin surface. The recorded signal represents a superposition of motor unit action potentials generated during muscle contraction, and its envelope reflects the overall activation level of the target muscle. Model-based estimation relies on a mathematical representation of the respiratory system to compute the total respiratory muscle pressure (*P*_mus_) from standard ventilator waveforms, including airway pressure (*P*_aw_), flow, and volume. From the reconstructed *P*_mus_ signal, commonly used effort metrics such as pressure–time product (PTP) and work of breathing (WOB) can be derived. Ultrasound imaging allows for the measurement of diaphragm thickness by positioning the probe in an intercostal space within the zone of apposition. Diaphragmatic contractile activity is quantified using the thickening fraction (TF_di_), calculated as the relative increase in diaphragm thickness from end-expiration to end-inspiration

This narrative review provides an integrative discussion of current approaches to noninvasively assess both the magnitude of the respiratory effort and its synchrony with the ventilator. We outline the physiological basis of these measurements, review potential methods—including surface EMG, model-based estimation, and ultrasound, as illustrated in Fig. 1—and discuss the critical steps needed for a broad clinical implementation.

## Methodology

This narrative review was conducted to provide a conceptual overview of noninvasive monitoring of respiratory drive and effort. Relevant literature was identified through non-systematic searches in PubMed and manual screening of reference lists. Articles were selected based on their relevance and contribution to current understanding. Findings are synthesized narratively to highlight key concepts, emerging evidence, and clinical implications.

## Respiratory effort

### Physiology

Ventilation is driven by the coordinated activation of multiple respiratory skeletal muscles. The diaphragm and intercostal muscles are the primary muscles under resting conditions, generating the negative intrathoracic pressure required for tidal breathing [10]. Under increased ventilatory demand, such as during exercise, respiratory distress, or pathological disease states, the accessory muscles, including the alae nasi, sternocleidomastoid, scalene, and genioglossus muscle, are recruited to augment inspiratory effort and maintain adequate ventilation [10]. Contraction of inspiratory muscles reduces pleural pressure, establishing a pressure gradient that facilitates air to flow into the lungs for gas exchange. The mechanical output resulting from this muscle activity reflects the pressure generated by the respiratory muscles and is referred to as *respiratory effort* [11–13].

### Measurements

As direct measurement of pleural pressure is virtually impossible to perform in routine clinical practice due to its invasiveness, esophageal pressure is often used as its surrogate. The pressure *P*_es_, as measured by esophageal balloon manometry, closely reflects intrathoracic pressure swings in the pleural space. From *P*_es_, the total output of all respiratory muscles *P*_mus_ can be estimated when corrected for the elastic pressure exerted by the chest wall. The pressure generated specifically by the diaphragm (*P*_di_) can be derived by measuring both *P*_es_ and gastric pressure (*P*_ga_), enabling analysis of the magnitude and timing of diaphragmatic effort. Commonly used metrics for quantifying effort include *work of breathing* (WOB) and *pressure–time product* (PTP), both of which are calculated directly from *P*_mus_ or *P*_di_ [8].

**Fig. 2:**
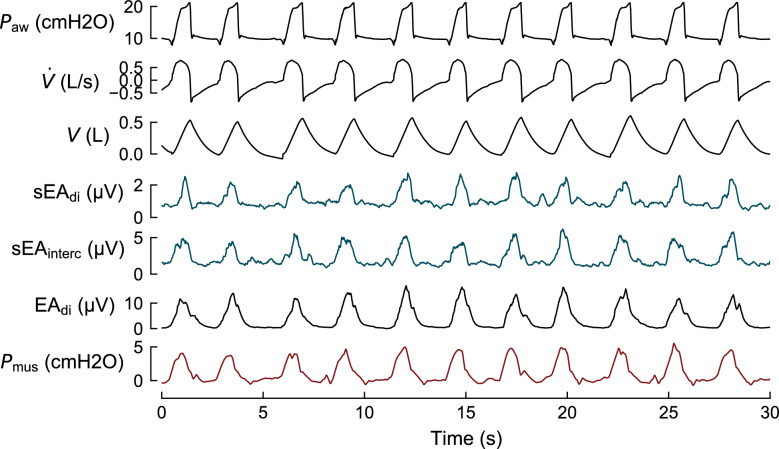
Airway pressure (*P*_aw_), flow ($\dot{V}$), volume (*V*), surface electromyography envelope signals of the diaphragm (sEA_di_) and intercostal muscles (sEA_interc_), invasively measured electrical activity of the diaphragm (EA_di_), and respiratory muscle pressure (*P*_mus_) derived from esophageal pressure (*P*_es_) in an intensive care patient undergoing assisted mechanical ventilation. Data from [16]

In clinical practice, the main alternative to measuring mechanical muscle output is the assessment of neural electrical activation. The *electromyogram* (EMG) provides a measure of motor unit action potentials during muscle activation—when recorded with electrodes on the skin overlying the muscle it is referred to as *surface EMG* (sEMG). In clinical practice, as the raw muscle EMG signal is difficult to interpret, the total *electrical activity* of a muscle is quantified via the amplitude (sometimes called envelope) of the EMG, which is shown in Fig. 2 for different muscles and measurement techniques. The *electrical activity* of the diaphragm (EA_di_) can be obtained invasively, using a specialized nasogastric catheter mounted with electrodes positioned in the gastroesophageal junction [9, 14], or noninvasively from sEMG recordings [15]. This noninvasive measure of electrical diaphragm activation termed *surface EA*_*di*_ (sEA_di_) can be easily applied in clinical practice and does not require long setup times. Figure 2 illustrates the described waveforms. When neural transmission is intact, the electrical activity of the diaphragm reflects the central activation from the respiratory center in both amplitude and timing. This neural transmission involves the generation of action potentials in the brainstem, which travel via the phrenic and other motor nerves to stimulate the respiratory muscles. As such, EA_di_ is often considered the closest available surrogate of the *neural respiratory drive*, defined as the electrical output from the brainstem [13], though, depending on the measurement setup, EA_di_ or sEA_di_ only capture a subset of motor units in the rural or costal part of the diaphragm. The relation between neural activation and mechanical output of a muscle is determined by its *neuromechanical coupling*, which measures how much pressure/force is generated per unit of electrical activity.

## Less invasive respiratory monitoring

With this review, we aim to contribute to the development and implementation of techniques for measuring respiratory effort that are easier to perform and interpret and less invasive than the nasogastric balloon catheter. Insertion of an esophageal or gastric pressure balloon has been referred to as both “minimally invasive” and “invasive” by different authors, though, the risk associated with inserting a nasogastric tube is certainly very limited, and only in rare cases serious complications have been reported [17, 18]. The main appeal of completely noninvasive methods for measuring effort is that they are usually less laborious in clinical practice and have much shorter preparation time than *P*_es_ measurements. Setting up the esophageal balloon is cumbersome, as it requires careful positioning and calibration of the catheter, without direct visual feedback. Proper positioning in the lower third of the esophagus is verified via the occlusion test [8], where the ratio of pressure changes in *P*_aw_ and *P*_es_ during an occluded inspiratory effort is typically targeted to be between 0.8 and 1.2. When spontaneous efforts are not present, manual chest compression during an expiratory hold is used to generate a deflection of *P*_aw_ and *P*_es_. The absolute value of *P*_es_ strongly depends on the filling volume of the balloon: at higher filling volumes, *P*_es_ has a substantial positive offset, which is caused by the elasticity of the esophageal wall, though a calibration procedure for the filling volume to deal with this baseline artifact has been proposed [19]. Yet, another practical difficulty of interpreting *P*_es_ is that a precise estimate of the elastic recoil of the chest wall is needed to calculate effort. The chest wall elastance can be measured during passive conditions, though in practice it is often approximated using the rule-of-thumb formula that compliance is 4% of the predicted vital capacity per cmH_2_O [8]. Moreover, the *P*_es_ signal is often strongly contaminated by cardiac artifacts and peristaltic esophageal contractions, which may complicate interpretation of the data. These time-consuming setups are especially problematic in understaffed wards, where qualified personnel are scarce. A simple, noninvasive technique will likely be more readily adopted than *P*_es_ and lead to more readily applied routine monitoring, allowing a larger patient population to benefit from a more comprehensive observation as a clinical default.

## From intermittent maneuvers to continuous monitoring

As summarized in previous literature reviews, occlusion maneuvers are currently the most used noninvasive measures to assess respiratory effort in daily clinical practice, with the highest degree of maturity, being available on all modern ventilators [5, 11, 12, 20]. The clinically widely used parameter *P*_0.1_ is defined as the drop of pressure in the first 100 ms of an inspiratory effort against occluded airways and reflects the neural output of the respiratory centers [21]. Telias and Spadaro [5] and de Vries et al. [11] recently provided important evidence for validating *P*_0.1_ as a standard tool for patients under assisted MV. They showed that *P*_0.1_ is correlated with measures of respiratory effort, including PTP/min (within-patients *r*^2^ ≥ 0.84). While it does enable detection of extreme respiratory effort (i.e., excessive and insufficient effort) with high sensitivity and specificity, it is far too artifact-prone to reliably measure exact values of respiratory effort [11]. An important technical factor influencing *P*_0.1_ is the difference in measurement procedure, inducing a bias if estimated without proper occlusions in certain ventilators [22]. Some ventilators perform an actual occlusion maneuver, whereas other devices extrapolate the value from the pressure drop during the triggering phase that is often shorter than 50 ms. Importantly, due to the average breath-to-breath variation within patients also turns out to be relatively high, with a coefficient of variation of up to 26% [22], which is why the common recommendation is to report averages over multiple occlusions.

The pressure *P*_occ_ is the maximal deflection from baseline during a fully occluded inspiratory effort. It was proposed independently from *P*_0.1_ as a surrogate for both inspiratory effort and lung stress [23, 24]. Compared to *P*_0.1_, *P*_occ_ exhibits reduced susceptibility to artifacts and greater discriminative power compared to *P*_0.1_, but is similarly unsuitable for precise effort quantification [6].

Although airway occlusions appear to be straightforward and widely accepted maneuvers, the major downside is their inherent discontinuity over time. Occlusion measures only represent the instantaneous respiratory effort at the very moment the maneuver is performed, often reported as average values over 3–5 single breaths. Continuous monitoring, on the other hand, enables the analysis of breath-to-breath variability, which is difficult to capture via intermittent measures. As such, increased work of breathing, signs of distress, or changes in respiratory patterns due to either changing disease status or ventilator settings can be promptly identified and addressed. Intermittent measures, moreover, only provide insight into the magnitude of respiratory drive and effort, but omit the timing and patient–ventilator interaction. Patient–ventilator asynchrony can only be deduced from a running ventilator with respiratory effort data generated during uninterrupted assisted MV.

In the following paragraphs, we discuss three noninvasive methods that provide a continuous estimate of the magnitude and timing of effort holding great promise to advance respiratory muscle monitoring, as presented in Fig. 1 and Table 1.

**Table 1:** Overview of noninvasive techniques for measuring respiratory effort

Noninvasive monitoring technique	Intermittent/continuous	Respiratory muscles	Best practices	Relation to effort (magnitude and asynchrony)	Required expertise	Clinical readiness	Challenges	Future perspectives
*P*_0.1_ and *P*_occ_	Intermittent	Total respiratory muscle output	To reduce noise, the average of 3–5 breaths should be reported	Magnitude: moderate to strong correlation [5]; both recognize the extremes of effort (excessive/insufficient) and both have high sensitivity to small efforts; *P*_occ_ has superior discriminative power than *P*_0.1_ Asynchrony: cannot be analyzed	Ventilator settings and interpretation of waveforms	Established in patient monitoring; widely available on mechanical ventilators	*P*_0.1_ is influenced by physiological and technical factors, including the performance of the occlusion itself and the specific algorithm; some ventilators perform an actual occlusion maneuver, others extrapolate the value from the triggering phase	The influence of ventilator models should be further investigated
Model-based	Continuous; lung parameters must be updated in regular intervals	Total respiratory muscle output	Lung parameters are estimated when the patient is passive or during maneuvers: a continuous *P*_mus_ signal is reconstructed from ventilator signals using the equation of motion	Magnitude: inconclusive: depending on the study, moderate or strong correlation has been reported [39–42] Asynchrony: one study [45] reported that the model-based method enabled detecting ineffective efforts as well as precise time of breath onsets/offsets	Respiratory mechanics and modeling, equation of motion	Experimental, but implementation available with the PAV+ ventilation mode	The standard equation of motion is not suited to represent nonlinear effects such as flow limitation	Models should be extended to reflect nonlinearity and confirmed on larger cohorts; model-based estimation will benefit from sensor fusion, e.g., integrating sEMG signals
sEMG	Continuous; long measurements > 1 h might be influenced by changes in fluid balance, electrode–skin contact	Diaphragm, intercostal, sternocleidomastoideus, scalene, abdominal wall muscles	Bilateral electrode positions: suppress ECG artifacts via gating, high-pass filtering, or wavelet denoising; neuromechanical coupling (NMC) measured with an end-expiratory occlusion maneuver	Magnitude: moderate to strong correlation [16, 27]; requires conversion via neuromechanical coupling (NMC) Asynchrony: major types detectable if SNR is sufficient	Electrode placement, skin preparation, interpretation of signals, waveforms and artifacts	Early clinical use, mostly research, lack of dedicated devices	Susceptible to electrical artifacts, crosstalk, motion interference; long-term stability not demonstrated yet	Further standardization, real-time processing, and longitudinal studies
Ultrasound	Continuous; thickening fraction relies on manual measurements; automated operator-independent device emerging	Diaphragm, intercostal, abdominal wall muscles	Thickening fraction of the diaphragm muscle is measured in the zone of apposition in the 8th–11th intercostal space; diaphragm thickening fraction (TF_di_) is calculated as the relative thickening during an inspiratory effort	Magnitude: moderate to strong correlation, not suitable to recognize precise values of effort [53, 54] Asynchrony: proposed [51, 59], but needs further research	Probe positioning; ultrasound image interpretation	Early clinical adoption; widely available in ICUs	Inter-observer variability	Automated image processing and analysis software; extra-diaphragmatic muscle imaging; AI-driven reproducibility

### Surface EMG for monitoring respiratory muscle activity

The electrical activity of the respiratory muscles can be measured both continuously and noninvasively by attaching electrodes to the skin overlying the respiratory muscles, as shown in Fig. 1. This technique is known as sEMG and captures the electrical surface potentials generated by the depolarization of muscle fibers during contraction [15]. Various electrode configurations can be used for the diaphragm, including unilateral and bilateral setups, which were systematically compared in a recent study [15, 25]. In contrast to the invasive method, surface electrodes also allow monitoring of the extra-diaphragmatic muscles, such as the parasternal intercostals, sternocleidomastoid, alae nasi, genioglossus, and scalene muscles, which are recruited when the diaphragmatic load is high [26].

**Fig. 3:**
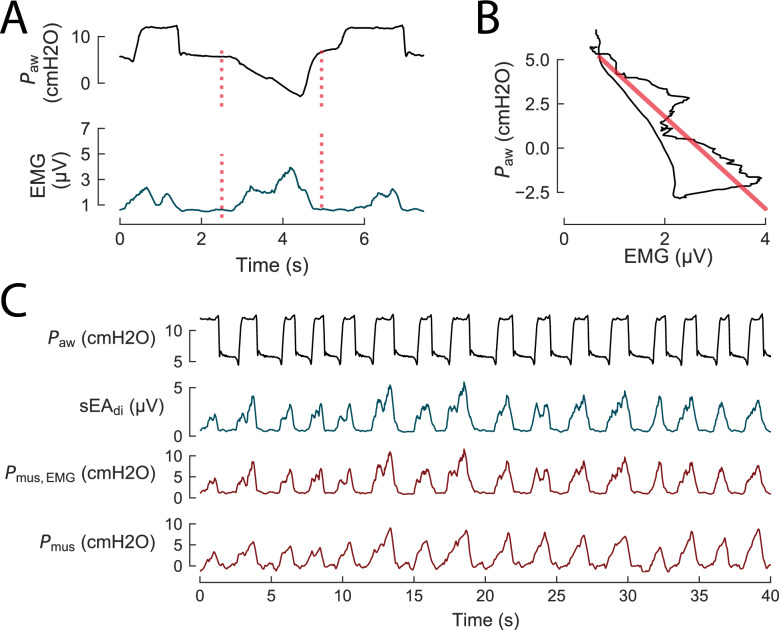
Estimation of respiratory effort from surface electromyography using neuromechanical coupling of the diaphragm (NMC_di_). Data are from a patient undergoing assisted mechanical ventilation [16]. **A** Airway pressure (*P*_aw_) and surface diaphragm EMG during an occlusion maneuver (between red dotted lines). **B** Relationship between *P*_aw_ and surface EMG during the occlusion. The slope of the linear regression (red line) represents the neuromechanical coupling of the diaphragm (NMC_di_). **C** NMC_di_ is used as a scaling factor to convert sEA_di_ into an estimate of respiratory effort during regular ventilation, providing a noninvasive surrogate (*P*_mus_, EMG) for respiratory muscle pressure (*P*_mus_) measured invasively with an esophageal balloon. Data from [16]

Surface EA_di_ (sEA_di_) is fundamentally a measure of neural respiratory drive, but it also serves as an index of respiratory effort. Both sEMG amplitude and area under the curve are shown in two smaller single-center studies to correlate near-linearly with respiratory effort as represented by *P*_mus_, across varying levels of ventilatory support (*r*^2^ ≥ 0.79) [16, 27]. This relationship is referred to as the neuromechanical coupling of the diaphragm (NMC_di_). When NMC_di_ is derived noninvasively through end-expiratory occlusion maneuvers, it can be used as a scaling factor. Translating the electrical activity (sEMG) into an estimate of the mechanical output (*P*_mus_) enables continuous and noninvasive monitoring of breath-by-breath respiratory effort [27–29], when ventilator settings remain stable. Figure 3 illustrates the methodological steps to use sEMG during an occlusion to calculate NMC_di_ and with that a continuous noninvasive estimate of *P*_mus_.

Beyond amplitude-based measures of effort, the timing of the sEA_di_ signal offers additional insights into patient–ventilator interaction. Surface EA_di_ provides a noninvasive method to assess the asynchrony between the patient’s neural respiratory drive and the mechanical support delivered by the ventilator, including delayed triggering, missed efforts, delayed cycling, double triggering, auto-triggering, and reverse triggering [30, 31].

#### Future perspectives of sEMG

The field of respiratory sEMG has seen substantial progress in recent years, particularly in the development of recording devices, signal processing techniques, and quality control measures. Yet, clinical adoption of sEA_di_ for continuous monitoring remains nevertheless limited due to several unresolved challenges regarding standardization of acquisition setups and signal processing. Surface EMG is not specific to a single muscle; thus, an important practical problem is crosstalk, e.g., from abdominal muscles, and between inspiratory and expiratory muscles. Optimized electrode positions have been shown to increase specificity to inspiratory muscles [25, 32]. The reliability of sEMG-derived metrics can also be improved through the introduction of quality criteria to identify non-physiological waveforms [29] or deriving signal quality indices to quantify the impact of disturbances, motion artifacts, and of crosstalk [30]. Another concern is the long-term stability of the sEA_di_ signal. Its inherently low voltage makes it susceptible to noise and fluctuations in measurement conditions. Factors such as diaphoresis, fluid balance, edema, electrode gel degradation, and patient movement can all compromise signal quality during a clinical course on the intensive care unit, potentially limiting its utility for longitudinal assessments [15, 29, 30]. In addition, intersubject variability and electrode placement complicate standardization across patients and sessions. While these challenges are well recognized, their clinical impact remains uncertain due to the absence of larger, longitudinal studies. Consequently, it is unclear whether these limitations represent minor technical hurdles or barriers to clinical adoption.

To transition from research to routine clinical use, standardization of acquisition protocols, signal pre- and post-processing, and interpretation frameworks are essential to overcome these aspects of longitudinal stability [15]. Future efforts should focus on developing robust, artifact-resistant sensors and automated quality control algorithms to ensure reliable signal capturing in inherently dynamic clinical environments. Ultimately, based on continued technological refinement and clinical research, sEA_di_ holds great promise as a noninvasive, continuous modality for monitoring respiratory drive and effort and guiding personalized respiratory care when supported by mechanical ventilation.

### Model-based estimation of respiratory effort

Computational physiological models (CPM) of the respiratory system represent individual pathophysiology and act as virtual patients to improve understanding of physiology, simulate data, or determine respiratory parameters, based on physics and mechanistic principles [15]. Estimation of respiratory effort based on these CPMs relies on fitting a lung mechanics model to the patient’s ventilator signals (airway pressure, volume, and flow), as illustrated in Fig. 1. Typically, the equation of motion ${\mathrm{Pmus}} = E \times V + R \times \dot{V} - {\mathrm{Paw}}$ is used as a basis to model the respiratory system. Under this model, *P*_mus_ is simply the pressure that remains after subtracting the ventilator support *P*_aw_ from the elastic recoil of the respiratory system (*E* × *V*) and the resistive pressure component ($R \times \dot{V}$) [15]. Thus, given values for the respiratory elastance *E* and airway resistance *R*, *P*_mus_ can be calculated directly from ventilator waveforms. The estimated *P*_mus_ signal, in theory, represents the total output of the respiratory pump, including contributions from both the diaphragm and accessory muscles.

A key challenge of the model-based approach is that respiratory parameters (*E* and *R*) cannot be easily estimated during spontaneous breathing, because the equation of motion does not have a unique solution for its parameters when *P*_mus_ ≠ 0 [33]. A first solution is to only estimate parameters when the patient is passive (*P*_mus_ = 0) and then reconstruct *P*_mus_ from these parameters when the patient is breathing spontaneously again. This approach has been successfully demonstrated in a few studies [34–36], albeit in small cohorts. Completely suppressing spontaneous breathing during parameter identification is often not desirable. An alternative is to estimate parameters when *P*_mus_ is assumed to be small (*P*_mus_≈0), e.g., during expirations or at high PS levels. Recently, two studies have also tested this approach, but as of now, results remain inconsistent with earlier studies on completely passive patients [37, 38]. Both Natalini et al. [37] and Ruiz Ferrón et al. [38] determined respiratory parameters under the highest pressure support levels but found that *P*_mus_ could not be accurately estimated. Still, the study by Natalini et al. showed that the model-based method can accurately identify patients with very low breathing effort.

Fewer attempts have been made to estimate lung parameters and *P*_mus_ simultaneously during assisted ventilation. An advanced approach is the one by Younes et al., estimating lung elastance and resistance by using maneuvers such as pressure pulses and occlusions [39, 40]. This method has been implemented with the commercially available PAV+ ventilation mode, where pressure is delivered in proportion to the estimated *P*_mus_ signal. Maneuvers facilitate parameter identification but inevitably disrupt the normal breathing rhythm. Thus, there is an inherent trade-off: the number of maneuvers should be minimized while still ensuring identifiability of the relevant model parameters. Younes et al. later proposed a variant of their approach, which does not rely on maneuvers but on simple heuristics for choosing parameters, and demonstrated that it still enabled analysis of patient–ventilator interaction [41]. This model-based method was validated against esophageal pressure [42] and showed good agreement with *P*_mus_ (*r* ≥ 0.77 for PTP values).

#### Future perspectives on model-based estimation

Many authors have recognized the potential of using computational physiological models for analyzing breathing effort, but so far, results have been inconclusive, i.e., different studies have reported widely varying levels of agreement to *P*_mus_ [34, 35, 37, 38, 42, 43]. The available studies are also relatively small and difficult to compare because they investigate different target metrics (WOB, PTP) and clinical cohorts. Some authors have urged caution regarding the capability of simple respiratory models to represent certain nonlinear lung mechanics, such as expiratory flow limitation or alveolar overdistension [44–46]. Model development inherently involves the deliberate selection of core physiological concepts to include while necessarily excluding others. As a result, no model can fully capture the complexity of clinical reality. However, absolute fidelity is not a prerequisite, as long as the model provides a sufficiently accurate approximation to support clinical decision-making at the bedside [47]. Future work in model-based effort estimation should investigate extensions of existing models that better account for relevant nonlinear characteristics.

**Fig. 4:**
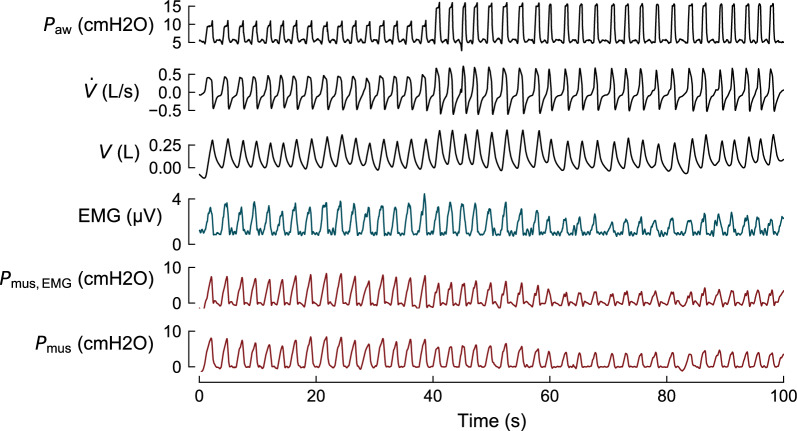
Model-based estimation of respiratory muscle pressure (*P*_mus_) using a hybrid model that uses both ventilator waveforms and surface EMG. The method combines airway pressure (*P*_aw_), flow ($\dot{V}$), volume (*V*), and sEMG to identify the parameters of a lung mechanics model algorithmically. Using these parameters, the respiratory muscle pressure (*P*_mus,EMG_) is reconstructed from ventilator data alone. The estimated *P*_mus_ closely approximates the invasively measured *P*_mus_ obtained from esophageal pressure recordings. Data and method previously published in [48]

A highly promising line of research is based on the idea of integrating additional (noninvasive) measurements into existing respiratory models, e.g., the integration of surface EMG, which has been proposed recently [48]. By combining multiple independent measurements in a single model, the resulting *P*_mus_ curve is less affected by noise in individual signals, and a highly accurate estimate can be attained. Figure 4 illustrates this approach: parameters of a lung mechanics model are identified by combining pneumatic signals with sEMG, which allows to reconstruct a *P*_mus_ signal from ventilator data alone.

To ensure a high level of utility, models must always undergo rigorous evaluation through verification, validation, and uncertainty quantification, collectively referred to as VVUQ [47]. Verification checks that the model works as intended, for example, by running simple tests and comparing results with known reference data. Validation asks: does the model reflect reality? This is done by comparing the model’s estimates of patient effort (*P*_mus_) with invasive measurements like esophageal pressure. Uncertainty quantification tells us how confident we can be in the model’s output. For instance, if lung compliance or resistance changes, the model should indicate how much this affects the predicted effort. Providing clinicians with an uncertainty measure alongside the estimated *P*_mus_ curve is essential for informed ventilator adjustments [49].

Despite the potential of model-based *P*_mus_ estimation, its practical usability in real clinical settings remains uncertain. Challenges include the difficulty of identifying patient-specific parameters under dynamic conditions, the impact of volume leaks and non-stationary breathing patterns on model accuracy, and the strong dependence on high-quality ventilator signals, which can be compromised by artifacts or sensor drift. Future work should focus on robust artifact handling and validation on larger cohorts to bridge the gap between theoretical accuracy and bedside feasibility.

### Ultrasound-based diaphragm monitoring

Ultrasound has been increasingly used in recent years to measure diaphragmatic anatomy and activity in critically ill patients, offering a noninvasive modality for continuous diaphragm monitoring. The main parameter derived from ultrasound that correlates with effort is the diaphragm thickening fraction during an active inspiration, measured in the zone of apposition [50]. The thickening fraction (TF_di_) can be determined in M-mode or B-mode as the ratio of tidal muscle thickening to muscle thickness at end-expiration:

$${\mathrm{TF}}_{{{\mathrm{di}}}} = \frac{{T_{{{\mathrm{ei}}}} - T_{{{\mathrm{ee}}}} }}{{T_{{{\mathrm{ee}}}} }} \cdot 100\% ,$$

where *T*_ei_ and *T*_ee_ denote diaphragm thickness at end-inspiration and end-expiration, respectively.

Values between 15 and 30% during assisted MV reflect adequate contractile activity of the diaphragm, being associated with stable muscle thickness and short duration of MV [51], and have been proposed as potential therapeutic targets for diaphragm protection during assisted ventilation. In a single-center study, TF_di_ had a strong correlation with measures of effort (*r* = 0.80 and *r* = 0.70 for PTP_es_ and PTP_di_, respectively) [52]. However, for a given effort level, there is a wide range of corresponding TF_di_ values across patients [52, 53], limiting its usefulness for precise respiratory effort monitoring, but enabling intra-patient trend monitoring. Importantly, diaphragm ultrasound requires substantial operator expertise, and reproducibility remains a concern. Inter-observer TF_di_ values differed by up to 16 percentage points (%pt) for marked probe sites and up to 27%pt for unmarked sites, indicating insufficient accuracy for the proposed 15–30% target range of [54]. This is currently one of the main barriers to the implementation of US for precise respiratory effort monitoring in clinical routines.

#### Future perspectives on ultrasound

Diaphragm ultrasound is a technique that could benefit greatly from automated image acquisition and AI-based processing software, e.g., for confirming correct positioning of the US transducer, segmenting the pleural and peritoneal membranes, or calculating a continuous signal of the diaphragm thickness. Pipeline automation might also help to improve the reproducibility of the technique. Speckle tracking ultrasound is hypothesized to offer a more precise and comprehensive assessment of diaphragm function by correlating with transdiaphragmatic pressure and EMG but requires similar automation and larger clinical studies to prove its value in respiratory effort assessment [55–57].

The assessment of patient–ventilator interaction with ultrasound has also been proposed using either diaphragm excursion (measured with M-mode from below the costal margin) or TF_di_ [50, 58], but still needs more extensive clinical validation. It is not yet clear whether long-term and continuous assessment of effort with ultrasound-derived TF_di_ is feasible in daily clinical practice. With current hand-held devices, positioning of the transducer requires manual adjustments, which impedes continuous measurements over longer periods of time. A promising continuous alternative has recently been demonstrated by Demoule et al. for measuring diaphragm excursion [59]. Their approach is based on a wearable ultrasound patch, which continuously measures the upper face of the liver as a proxy for the diaphragm.

Ultrasound of extra-diaphragmatic muscles has been proposed, e.g., for the parasternal intercostal muscles, as well as abdominal wall muscles, but the relation of their thickening fractions to respiratory effort needs more in-depth investigation. Further developments might be expected from shear wave elastography, an innovative ultrasound technique that assesses biomechanical properties of tissues by quantifying the shear wave speed generated by a focused ultrasound beam [60]. When applied to the diaphragm, this approach may offer new insights into structural changes, although, again, clinical trials are required to better understand the utility of this method.

## Discussion

Noninvasive and continuous monitoring of respiratory effort holds tremendous potential for advancing patient monitoring. Compared to nasogastric catheter-based techniques, noninvasive methods are less labor-intensive and easier to implement at the bedside, potentially enabling wider application. While occlusion-based techniques for now remain the most accessible tools in current practice, their inability to capture continuous effort and patient–ventilator asynchrony limits their utility for monitoring the impact of muscle effort. The three noninvasive and continuous methods for respiratory effort assessment discussed in this review—surface EMG, model-based estimation, and ultrasound—enable continuous assessment of both the magnitude and timing of respiratory muscle activity. These advances should facilitate broader adoption of diaphragm-protective ventilation strategies in patients receiving assisted mechanical ventilation.

We compare challenges and future perspectives of all methods in Table 1. Of the three emerging methods, ultrasound is currently the most widely available in ICUs. It offers immediate, muscle-specific insight into the respiratory pump (including extra-diaphragmatic contributions), but it is not able to precisely quantify muscle effort. For analyzing asynchrony, surface EMG appears to be the most promising candidate, because, in contrast to ultrasound, it detects the precise timing of respiratory efforts. Long-term measurements remain a concern in both methods, but a first operator independent, wearable ultrasound device has been introduced. Model-based methods are still more experimental and quantify the total output of the respiratory pump. As they only rely on ventilator signals, they are intrinsically suited to long-term monitoring and less dependent on noise than ultrasound and sEMG. Combining multiple noninvasive techniques, such as integrating surface EMG with occlusion maneuvers or model-based estimation, holds promise for enhancing robustness and clinical applicability.

Several limitations of this review must be acknowledged. Many intensive care studies investigating innovative measurement techniques are relatively small, with only a few dozen subjects or less. This also concerns many of the results discussed in this review, i.e., most of the cited publications are based on small, single-center cohorts. Conclusions drawn from these studies come with uncertainty and must be confirmed externally on larger cohorts. Moreover, the studies discussed and presented in this review were selected to highlight important concepts, but this does not replace a systematic literature analysis. As this is a narrative review, there is an inherent danger of reporting bias, e.g., due to conflicts of interest, which we have disclosed in detail in the appendix.

## Data Availability

No datasets were generated or analysed during the current study.

## Abbreviations

MV: Mechanical ventilation
*P*_0.1_: Occlusion pressure after 100 ms
*V*: Lung volume
$$\dot{V}$$: Airway flow
*P*_aw_: Airway pressure
*P*_occ_: Pressure drop of an occluded breath
*P*_es_: Esophageal pressure
*P*_mus_: Respiratory muscle pressure
EA_di_: Electrical activity of the diaphragm
sEA_di_: Surface electrical activity of the diaphragm
sEA_interc_: Surface electrical activity of the intercostal muscles
EMG: Electromyography/electromyogram
ECG: Electrocardiography/electrocardiogram
sEMG: Surface electromyography/electromyogram
*P*_mus,EMG_: Respiratory muscle pressure derived from EMG
*P*_di_: Transdiaphragmatic pressure
*P*_ga_: Gastric pressure
PTP: Pressure–time product
PTP_di_: Diaphragmatic pressure–time product
PTP_es_: Esophageal pressure–time product
WOB: Work of breathing
NMC: Neuromechanical coupling
NMC_di_: Neuromechanical coupling of the diaphragm
PAV+: Proportional assist ventilation
TF_di_: Thickening fraction of the diaphragm
VILI: Ventilator induced lung injury
VIDD: Ventilator induced diaphragm injury
VVUQ: Verification, validation, uncertainty quantification
